# Influence of Caspase-9 polymorphisms on the development of Chronic Myeloid Leukemia- A case-control study

**DOI:** 10.1016/j.gene.2018.100002

**Published:** 2018-12-17

**Authors:** Prajitha Mohandas Edathara, Manjula Gorre, Sailaja Kagita, Anuradha Cingeetham, Sandhya Annamaneni, Raghunadharao Digumarti, Vishnupriya Satti

**Affiliations:** a Department of Genetics, Osmania University, Hyderabad, Telangana, India; b Department of Biochemistry, Osmania University, Hyderabad, Telangana, India; c Homi Bhabha Cancer Hospital and Research Centre, Visakhapatnam, Andhra Pradesh, India; d Jawaharlal Nehru Technological University (JNTU), Hyderabad, Telangana, India

**Keywords:** CML, Chronic Myeloid Leukemia, CASP9, Caspase 9, NIMS, Nizams Institute of Medical Sciences, RFLP, Restriction Fragment length Polymorphism, BCR-ABL, Breakpoint Cluster Region-Abelson, APAF1, apoptotic protease activating factor 1, BCL2, B-cell lymphoma 2, SNPs, single nucleotide polymorphisms, Sp1, simian virus-40 Protein 1, (EGR2 also termed Krox20) - Krox-20, early growth response protein 2, NF-I, nuclear factor I, ETF, EGFR-specific transcription factor, Ph, Philadelphia, WBC, white blood cells, EUTOS, EUropean Treatment Outcome Study, IM, Imatinib mesylate, EFS, event free survival, OS, overall survival, PCR, polymerase chain reaction, CCgR, complete cytogenetic response, SAS, South Asian, EAS, East Asian, HCB, Asian, CHB, Asian, JPT, Asian, AMR, Ad Mixed American, EUR, European, CEU, European, AFR, African, YRI, Sub-Saharan African, HWE, Hardy Weinberg Equilibrium, LD, linkage disequilibrium, Asp, aspartate, AML, acute myeloid leukemia, Apoptosis, Chronic Myeloid Leukemia, Caspase 9 polymorphisms, RFLP, Haplotype

## Abstract

**Introduction:**

Chronic Myeloid Leukemia (CML) is a myeloproliferative disorder, characterized by the overproduction of myeloid cells in all stages of maturation. It is usually defined by three sequential stages (Chronic, Accelerated and Blast-crisis) where the transition from chronic to accelerated to blast phases is presumed to occur due to secondary genetic changes, viz. accumulation of mutations, activation of downstream pathways and failure of apoptosis. Caspase 9 is the initiator caspase involved in mitochondrial-mediated apoptotic pathway. Polymorphisms in the promoter (−1263 A>G, -712C>T, -293 del) and coding (Ex5 +32G>A) regions of CASP9 gene are found to influence the expression levels by either impairing the activation or loss of expression of CASP9 or insufficient formation of apoptosome.

**Methods:**

The present case-control study was carried out on 999 individuals, comprised of 485CML cases reported at Nizams Institute of Medical Sciences (NIMS), Hyderabad and 514 age and gender-matched healthy individuals from local population. DNA was isolated by non-enzymatic/salting-out method and was genotyped using RFLP technique.

**Results:**

It was observed that the presence of G allele of CASP9 -1263A>G polymorphism enhanced the risk for CML while CASP9 -712C>T and CASP9 -293del SNPs conferred protection against development of CML. Haplotype analysis of promoter and exonic polymorphisms had revealed increased risk associated with two haplotypes G_C_del (+)_G (OR-1.61, 95% CI-0.97-2.65, p-0.06#) and G_C_del (–)_G (OR-2.09, 95% CI-0.94-4.66, p-0.07#) suggesting the role of G allele of CASP9 -1263A>G in conferring risk independent of other SNPs. Pairwise LD analysis performed for all the four SNPs revealed the presence of LD among the SNPs.

**Conclusion:**

The results of the present study therefore concludes the role of CASP9 -1263A>G polymorphism in increasing the risk for the development and progression while CASP9 -712C>T and CASP9 -293del SNPs conferred protection and CASP9 Ex5 +32G>A was involved in conferring resistance which could be in combination with other SNPs or factors affecting them.

## 1 Introduction

CML is a hematological malignancy, characterized by the abnormal proliferation of myeloid progenitors of hematopoietic system. It originates from the transformation of a single hematopoietic cell which carries the BCR-ABL translocation that escapes the cell surveillance mechanism. The presence of Bcr-Abl drives the clonal expansion of leukemic progenitor cells which is mainly dependent upon the imbalance between the rate of proliferation and rate of cell death. The cells expressing Bcr-Abl therefore, prolongs the growth factor-independent survival by inhibiting apoptotic machinery (Bedi et al., 1994). Bcr-Abl oncoprotein was found to exert its anti-apoptotic effect both directly and indirectly by deregulating the genes involved in the apoptotic machinery through aberrant expression of anti- and pro-apoptotic genes. This results in the inhibition of release of cytochrome *c* from the mitochondria and subsequent activation of Apaf1 which are found to inhibit the activation of Caspase 9 (Cai et al., 1998).

Caspase 9 is an initiator caspase which is mainly involved in the intrinsic/mitochondrial mediated apoptotic pathway. Activation of Caspase 9 is often linked to the permeabilization of outer mitochondrial membrane by pro-apoptotic members of BCL2 family. SNPs located either in the promoter region (CASP9-1263A>G, CASP9 -712C>T, CASP9 -293 del) or in the exonic region (CASP9 Ex5 +32G>A) of Caspase 9 are known to modulate the susceptibility to cancer. In silico analysis (Park et al., 2006) had revealed creation of additional simian virus-40 protein 1- (Sp1) binding site in presence of CASP9 -1263G allele and elimination of binding sites for Krox-20, NF-1, and ETF transcription factors in the presence of CASP9 -712T allele, while the deletion of 19 (-293_-275 del CGTGA GGTCAGTGCGGGGA) nucleotides was reported to alter the expression levels of CASP9 gene. CASP9 Ex5 +32G>A exonic polymorphism results in the substitution of glutamine by arginine at codon 221 (Q221R). This amino acid substitution leads to conformational change in the molecule, which modifies the affinity of Casp9 protein to Apaf-1, thus influencing apoptosis and thereby carcinogenesis (Hirano et al., 2001). Keeping in view the functional significance of the promoter and exonic SNPs of CASP9 gene, the study was planned to assess the role of these SNPs in the development and progression of CML.

## 2 Materials and methods

### 2.1 Study design

The present case-control study was performed on 999 individuals comprising of 514 controls and 485 CML cases. Age and gender matched control samples without any family history of cancer were obtained from the local population, while the CML cases were recruited from Nizams Institute of Medical Sciences (NIMS), Hyderabad. This study was approved by the Institutional Ethics Committee for Biomedical Research, Osmania University, and ethical committee of the NIMS, Hyderabad. CML patients with primary diagnosis of Ph + ve CML, who are being treated with Imatinib mesylate (IM), irrespective of age group were recruited, while secondary or drug induced CML cases were excluded. The diagnosis of the CML was confirmed based on the complete blood picture (WBC, platelets and % of blasts), cytogenetic tests (to detect the presence of Philadelphia chromosome in blood and bone marrow) and molecular analysis (to detect the levels of BCR-ABL transcript). All the patients who participated in the study were informed about the objectives and a written informed consent was obtained from them. The patient's gender, age at the time of diagnosis, occupation and other general information was noted down in prescribed proforma. The clinical information such as- Baseline clinical profile (WBC count, platelet count, % of blast and differential cell count (basophils, eosinophils) and spleen size), Phase of the disease at the time of diagnosis (chronic/accelerated/blast), Hematological response (responders or non-responders), Cytogenetic response (responders or non-responders) and Molecular response (responders or non-responders) was obtained from the tumor registry (Edathara et al., 2016). Three different risk scores were calculated namely Sokal, Hasford and EUTOS scores, using an online calculator http://bloodref.com/myeloid/cml/sokal-hasford).

IM resistance was categorized either as primary and secondary resistance based on specific criteria as follows. The patients who failed to achieve initial cytogenetic response (at six months) with Imatinib treatment were categorized into primary resistant cases, while patients who initially responded well to IM, but later tend to develop resistance during the course of the treatment were categorized into secondary resistant cases. Any indication of progression or death in chronic phase patients was considered as an event and the Event free survival (EFS) was calculated by taking the time lapsed from the date of diagnosis to the date of either entering into advanced phase (accelerated or blast) or death. All the patients were followed up for a period of five years and the percentage of patients who survived beyond 5 years after diagnosis was calculated and referred as relative five year overall survival (OS).

### 2.2 Methodology

Blood samples collected in EDTA were used for genomic DNA isolation by conventional salting out method (Lahiri and Nurnberger, 1991). The purity and concentration of genomic DNA was determined using Nanodrop (TM) 1000 UV/VIS Spectrophotometer (Thermo Fisher Scientific, Waltham, USA). The DNA samples were diluted to 50 ng/μL concentration. PCR was performed independently for four SNPs (CASP9 -1263A>G, CASP9 -712C>T, CASP9 -293 del and CASP9 Ex5 +32G>A) following the PCR conditions which included initial denaturation at 95 °C for 5 min, followed by 35 cycles of denaturation at 94 °C for 1 min, annealing at varied temperature (based on SNP), extension at 72 °C for 1 min and final extension at 72 °C for 7 min using sequence specific primers (BIOSERVE) (Table 1).

**Table 1:** PCR conditions used for SNP analysis.

Gene, chromosome location, base change, position	Genotyping method	Primer	Annealing temperature	PCR Product
CASP9 (1p36.21); -1263A>G; rs4645978	PCR RFLP	F:5′-GGGAATACTTTCCTGGCAGG-3′ (sense) R:5′-GTCTTCCATTCCCTCTTCCG(C/G)TC-3′ (antisense)	59.8 °C	234 bp
CASP9 (1p36.21); -712C>T; rs4645981	PCR RFLP	F:5′-AGTCGCGGAGGTGCCGCCTT-3′ (sense) R:5′-AGGGCTAGCCTCGTGCCAG(C/G)C-3′ (antisense)	62.5 °C	194 bp
CASP9 (1p36.21); -293 del; rs4645982	PCR assay (Hot Start Taq pol)	F:5′-CGTTGGAGATGCGTCCTGCG-3′ (sense) R:5′-CGCCCTCAGGACGCACCTCT-3′ (antisense)	64.5 °C	241 bp(++) 222 bp (−−)
CASP9 (1p36.21) Ex5 +32G>A; rs1052576	PIRA-PCR: mismatch, sense primer −2 G-to-C	5′-GGCTTTGCTGGAGCTGGCCC-3′ (sense) 5′-AGTACCCAATGCCTGCCCAGGG-3′ (antisense)	62.8 °C	121 bp

The PCR master-mix was prepared in a 10-μL reaction mix that consisted of 50 ng DNA template, 2.5 mM dNTP mix, 25 pmol of sense and antisense primers, 0.5 U Taq polymerase (Bangalore Genei) and Milli-Q water. PCR products were checked on 3% agarose gel and the products were digested overnight for 16 h and incubated at 37 °C in the water bath using specific restriction digestion enzymes based on SNP (Table 2). Genotyping of the samples was carried out on 4.5% agarose gel and the sizes of the fragments were determined with the help of DNA ladders (50 bp and 100 bp). Some of the samples were randomly genotyped and the results were found to be concordant.

**Table 2:** Genotyping of CASP9 polymorphisms after restriction digestion.

Gene & base change, position	Genotyping method	Enzyme (New England Biolabs)	Gel band pattern
CASP9 (1p36.21); -1263A>G; rs4645978	PCR RFLP (Taq polymerase)	BcoD1 (37 °C)	AA:205 bp,29 bp; AG: 234 bp, 205 bp, 29 bp; GG: 234 bp
CASP9 (1p36.21); -712C>T; rs4645981	PCR RFLP (Taq polymerase)	*Hae*II (37 °C)	CC:176 bp,18 bp; CT:194 bp,176 bp, 18 bp; TT 194 bp
CASP9 (1p36.21); -293 del; rs4645982	PCR assay (Hot Start Taq polymerase)	–	+/+:241 bp; −/+:241 bp,222 bp; −/−: 222 bp
CASP9 (1p36.21) Ex5 +32 G>A; rs1052576	PIRA-PCR: mismatch, sense primer −2 G-to-C (Taq polymerase)	*Msp*I (37 °C)	GG:102 bp, 19 bp; GA:121 bp, 102 bp, 19 bp; AA: 121 bp

### 2.3 Statistical analysis

Appropriate statistical analyses were performed to assess the association of CASP9 polymorphisms with CML. An online web tool, SNPSTAT, (http://bioinfo.iconcologia.net/snpstats/start.htm) was used to calculate the genotype frequencies, Hardy Weinberg Equilibrium (HWE) and odds ratios. The allele frequencies and chi-square p-values were calculated using online statistical tools (http://www.had2know.com/academics/hardy-weinberg-equilibriumcalculator-2alleles.html and http://www.quantpsy.org/chisq/chisq.htm). The level of significance was taken as p < 0.05. Haplotype and pairwise linkage disequilibrium analyses were derived using Haploview (version 4.1). SPSS version 22 software was used for analyzing the association of genotype with survival rates (Event free survival and Five year overall survival).

## 3 Results

### 3.1 Distribution of epidemiological and clinical variables in CML

In the present study, elevated sex ratio was observed in CML group wherein the frequency of male patients was found to be twice that of females. Major proportion of CML cases was in middle age group (20–40 years) and the overall mean age at onset was found to be 35.86 ± 12.37 years. It was also observed that the higher frequency of CML patients to be agricultural laborers or general laborers and more frequently non-vegetarians. However, area of living (rural or urban) did not show any association with CML (Table 3).

**Table 3:** Distribution of epidemiological variables in CML cases and controls.

Characteristic		Controls N (%)	CML Cases N (%)	Mean age at onset ± SD	Sex ratio
Gender	Male	309 (60.12)	312 (64.33)	36.03 ± 12.17	1.80:1
	Female	205 (39.88)	173 (35.67)	35.55 ± 12.74	
Age at onset	<20 yrs	61 (11.87)	36 (7.42)	15.08 ± 4.13	1.4:1
	20–40 yrs	278 (54.09)	295 (60.82)	30.74 ± 5.90	1.81:1
	>40 yrs	175 (34.04)	154 (31.75)	50.53 ± 6.66	**1.91:1**
Living area	Rural	280 (57.14)	278 (58.77)	35.58 ± 11.76	**1.96:1**
	Urban	210 (42.86)	195 (41.23)	35.82 ± 13.15	1.5:1
Occupation	Agriculture	26 (5.53)	94 (19.79)	37.88 ± 11.17	2.48:1
	Laborers	122 (25.96)	151 (31.79)	35.79 ± 10.85	**2.68:1**
	Others	322 (68.51)	230 (48.42)	34.77 ± 13.64	1.21:1
Diet	Veg	78 (16.15)	25 (5.57)	42.84 ± 13.84	1.5:1
	Non-veg	405 (83.85)	424 (94.43)	35.47 ± 12.31	**1.75:1**

CML cases: Mean age = 35.86 ± 12.37; SEM = 0.56; median = 35 (4–80).

There was a significant elevation in mean levels of WBC, platelet count, Sokal score, Hasford score and EUTOS score with large standard deviation and wide range of values indicating the presence of inter-individual variation. It was observed that 21.49% of CML patients failed to achieve complete cytogenetic response (CCgR). The mean event free survival rate in patients, diagnosed in chronic phase was found to be 69.52 months and relative five year overall survival was found to be 49.51 months (Table 4). Higher frequency of CML patients was observed in high risk group of Sokal (38.43%), intermediate group of Hasford (42.79%) and low risk group of EUTOS (64.19%). Although, the sex ratio was nearly twice in general among all groups, it was found to be more predominant in low and intermediate risk groups of Sokal (1.90:1) and intermediate risk group of Hasford (1.8:1) scoring systems and high risk group (1.78:1) of EUTOS scoring system. Nearly, 90% of patients were diagnosed in the chronic phase as compared to 6.6% and 3.4% of the patients reported in accelerated and blast phase, respectively. With respect to IM response, it was observed that nearly, 74.79% of patients achieved complete hematological response, 63.35% of the patients achieved complete cytogenetic response and 57.89% of the patients achieved complete molecular response (Table 5).

**Table 4:** Baseline clinical characteristics of CML patients.

Variable	N	Mean ± SD	Range
WBC count	461	142,368.51 ± 97,451.86	1100–740,000
Platelet count (in lakhs)	457	3.96 ± 2.43	0.3–20
Sokal score	458	3.55 ± 45.88	0.44–982.35
Hasford score	458	1085.76 ± 607.66	0–5260
EUTOS score	458	74.27 ± 46.05	0–277
% of no CCgr (%)	457	21.49 ± 8.16	11–66
Event free survival (months)	239	69.52 ± 34.70	1–170
Relative overall survival (months)	458	49.51 ± 18.53	1–61

**Table 5:** Distribution of clinical characteristics of CML patients.

Clinical variables		N (%)	Mean age ± SD	Sex ratio
Sokal score (458)	Low risk	134 (29.26%)	32.53 ± 11.36	1.85:1
	Intermediate risk	148 (32.31%)	35.66 ± 12.32	1.90:1
	High risk	176 (38.43%)	37.71 ± 12.78	1.55:1
Hasford score (458)	Low risk	166 (36.24%)	34.00 ± 9.67	1.68:1
	Intermediate risk	196 (42.79%)	34.96 ± 13.60	1.8:1
	High risk	96 (20.96%)	39.35 ± 13.28	174:1
EUTOS score (458)	Low risk	294 (64.19%)	36.16 ± 12.29	1.70:1
	High risk	164 (35.81%)	34.41 ± 12.51	1.78:1
Phase of CML (470)	CP	423 (90%)	35.63 ± 12.34	1.78:1
	AP	31 (6.60%)	36.13 ± 13.01	2.1:1
	BC	16 (3.40%)	31.75 ± 9.33	1.67:1
Hematologic response (365)	CHR	273 (74.79%)	35.47 ± 11.76	1.76:1
	PHR	56 (15.34%)	35.21 ± 10.88	1.95:1
	NHR	36 (9.86%)	35.50 ± 10.66	2:1
Cytogenetic response (352)	CCR	223 (63.35%)	35.17 ± 12.12	1.65:1
	PCR	63 (17.90%)	33.08 ± 10.31	2.15:1
	NCR	66 (18.75%)	35.70 ± 10.80	1.64:1
Molecular response (323)	Responders	187 (57.89%)	35.88 ± 12.03	1.67:1
	Non-responders	136 (42.11%)	34.82 ± 11.94	1.57:1

### 3.2 Genotype distribution of CASP9 polymorphisms in CML

**Fig. 1:**
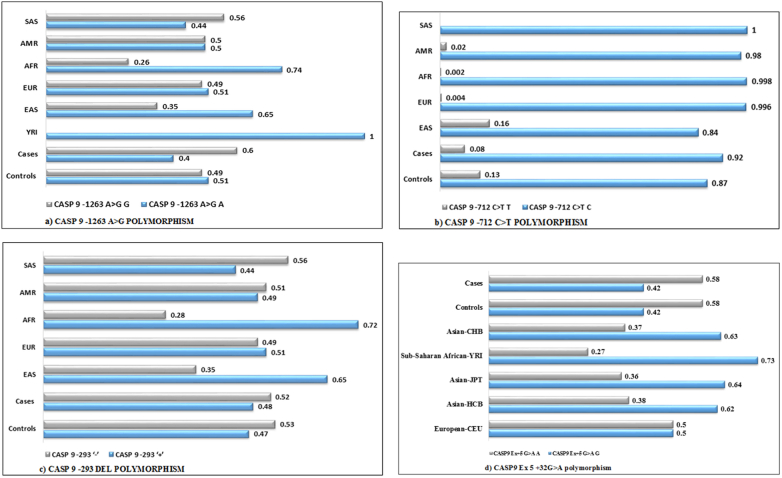
Hapmap data of Caspase 9 polymorphisms.

HapMap was constructed in order to compare the distribution of the allele frequencies of different SNPs of CASP9 under study with those reported from different populations like South Asian (SAS), East Asian (EAS), Asian (HCB), Asian (CHB), Asian (JPT), Ad Mixed American (AMR), European (EUR), European (CEU), African (AFR) and Sub-Saharan African (YRI). It was observed that the distribution of the variant allele frequencies of CASP9 -1263A>G and CASP9 -293 del polymorphisms were comparable to South Asian (SAS) population and Ad Mixed American (AMR) and European (EUR- 0.49) populations, respectively while variant allele frequency of CASP9 -712C>T polymorphism was greater than Ad Mixed American (AMR), African (AFR) and European (EUR) populations whereas CASP9 Ex5 +32G>A was greater than that of other populations like European (CEU), Asian (HCB), Asian (CHB), Asian (JPT) and Sub-Saharan African (YRI) (Fig. 1).

Table 6 shows the distribution of genotype and allele frequencies of CASP9 -1263A>G, CASP9 -712C>T, CASP9 -293 del and CASP9 Ex5 +32G>A polymorphisms among controls and cases. The allele distribution of CASP9 -712C>T and CASP9 -293 del polymorphisms had shown significant deviation from HWE whereas CASP9 Ex5 +32G>A polymorphism was in HWE in both controls and cases, while CASP9 -1263A>G polymorphism had shown significant deviation from HWE in controls but not in cases. The possible reason for deviation from HWE could be due to selective forces acting against or in favor of either of these alleles or due to elevated frequency of heterozygotes compared to both homozygotes (Table 6). It was observed that presence of either CASP9 -1263AG or CASP9 -1263GG genotypes was significantly associated with increased risk to develop CML under co-dominant, dominant and recessive models, respectively, with corresponding increase in the frequency of G allele. Conversely, the CASP9 -712CT genotype/T allele of CASP9 -712C>T polymorphism significantly conferred reduced risk for the development of CML under codominant, dominant and over-dominant models. With respect to CASP9 -293 del polymorphism, the frequency of heterozygous (+/−) genotype was elevated among controls as compared to cases and was associated with reduced risk for CML development under co-dominant, dominant and over-dominant models. However, the allele frequency distribution of CASP9 -293 del polymorphism did not reveal any significant difference between cases and controls. The present study, however, failed to reveal significant association of CASP9 Ex5 +32G>A polymorphism with CML development (Table 6).

**Table 6:** Distribution of CASP9 -1263 A>G polymorphisms among CML cases and controls.

Model	Genotype	Control	Case	OR (95% CI)	p-Value
*CASP9 -1263A>G (Hardy Weinberg Equilibrium: controls p = 0.00028; cases p = 0.78)*					
Codominant	AA	151 (29.61%)	79 (16.4%)	1.00	<0.0001*
	AG	214 (41.96%)	229 (47.4%)	**2.03 (1.46–2.83)**	
	GG	145 (28.43%)	175 (36.2%)	**2.28 (1.61–3.24)**	
Dominant	AA	151 (29.61%)	79 (16.4%)	1.00	<0.0001*
	AG + GG	359 (70.4%)	404 (83.6%)	**2.13 (1.57–2.90)**	
Recessive	AA+AG	365 (71.6%)	308 (63.8%)	1.00	0.01*
	GG	145 (28.43%)	175 (36.2%)	**1.42 (1.08–1.86)**	
Overdominant	AA+GG	296 (58%)	254 (52.6%)	1.00	**0.08**^**#**^
	AG	214 (42%)	229 (47.4%)	**1.25 (0.97–1.61)**	
Wild allele	A	516 (50.59%)	387 (40.06%)	1.00	
Variant allele	G	504 (49.41%)	579 (59.94%)	**1.54 (1.29–1.84)**	<0.0001*

*CASP9 -712C>T (Hardy Weinberg Equilibrium: controls p ≤0.0001; cases p ≤0.0001)*					
Codominant	CC	407 (80.12%)	432 (89.6%)	1.00	1e−04*
	CT	69 (13.58%)	27 (5.6%)	**0.38 (0.24–0.60)**	
	TT	32 (6.30%)	23 (4.8%)	0.67 (0.38–1.16)	
Dominant	CC	407 (80.1%)	432 (89.6%)	1.00	<0.0001*
	CT+TT	101 (19.9%)	50 (10.4%)	**0.47 (0.33–0.68)**	
Recessive	CC+CT	476 (93.7%)	459 (95.2%)	1.00	**0.26**
	TT	32 (6.30%)	23 (4.8%)	0.73 (0.42–1.27)	
Overdominant	CC+TT	439 (86.4%)	455 (94.4%)	1.00	<0.0001*
	CT	69 (13.6%)	27 (5.6%)	**0.39 (0.24–0.62)**	
Wild allele	C	883 (86.91%)	891 (92.43%)	1.00	
Variant allele	T	133 (13.09%)	73 (7.57%)	**0.55 (0.40–0.74)**	0.0001*

*CASP9 -293 DEL (Hardy Weinberg Equilibrium: controls p = 0.061; cases p ≤0.0001)*					
Codominant	+/+	121 (23.87%)	140 (29.3%)	1.00	0.03*
	+/−	231 (45.56%)	180 (37.7%)	**0.67 (0.49–0.92)**	
	−/−	155 (30.57%)	158 (33%)	0.88 (0.63–1.22)	
Dominant	+/+	121 (23.87%)	140 (29.3%)	1.00	0.05*
	(+/−) + (−/−)	386 (76.1%)	338 (70.7%)	**0.75 (0.57–1.00)**	
Recessive	(+/+) + (+/−)	352 (69.4%)	320 (67%)	1.00	0.41
	−/−	155 (30.6%)	158 (33%)	1.12 (0.86–1.47)	
Overdominant	(+/+) + (−/−)	276 (54.4%)	298 (62.3%)	1.00	0.01*
	+/−	231 (45.6%)	180 (37.7%)	0.72 (0.56–0.93)*	
Wild allele	+	473 (46.65%)	460 (48.12%)	1.00	
Variant allele	−	541 (53.35%)	496 (51.88%)	0.94 (0.79–1.13)	0.52

*CASP9 Ex5 +32G>A (Hardy Weinberg Equilibrium: controls p = 0.24; cases p = 0.26)*					
Codominant	GG	83 (16.40%)	78 (16.5%)	1.00	1
	GA	261 (51.58%)	244 (51.5%)	0.99 (0.70–1.42)	
	AA	162 (32.12%)	152 (32.1%)	1.00 (0.68–1.46)	
Dominant	GG	83 (16.4%)	78 (16.5%)	1.00	0.98
	GA+AA	423 (83.6%)	396 (83.5%)	1.00 (0.71–1.40)	
Recessive	GG+GA	344 (68%)	322 (67.9%)	1.00	0.99
	AA	162 (32%)	152 (32.1%)	1.00 (0.77–1.31)	
Overdominant	GG+AA	245 (48.4%)	230 (48.5%)	1.00	0.98
	GA	261 (51.6%)	244 (51.5%)	1.00 (0.77–1.28)	
Wild Allele	G	427 (42.19%)	400 (42.19%)	1.00	
Variant Allele	A	585 (57.81%)	548 (57.81%)	1.00 (0.84–1.20)	0.99

OR- odds ratio adjusted by age and sex; p value- χ^2^ p value; *p < 0.05; ^#^p < 0.10.

### 3.3 Association of CASP9 polymorphisms with phase of CML

When the data was compared with respect to the phase of the CML at the time of diagnosis, it was observed that the frequencies of AG and GG genotypes of CASP9 -1263 A>G polymorphism, in general, were significantly elevated in CML patients irrespective of phase at the time of diagnosis and significant odds ratios were obtained for chronic phase CML patients carrying either AG (OR-1.99, 95% CI-1.41–2.80) or GG (OR-2.20, 95% CI-1.53–3.16) genotypes. When the data on chronic phase CML was further stratified based on their progression as observed during follow up, it was noted that risk conferred by AG or GG was more pronounced for those who progressed into advanced phase (AG- OR- 2.43, 95% CI- 1.4–3.97; GG- OR- 2.46, 95% CI-1.46–4.14) as compared to patients who did not progress (AG- OR- 1.75, 95% CI - 1.17–2.61; GG- OR- 2.06, 95% CI-1.35–3.14) suggesting the role of this polymorphism in progression of CML. In case of CASP9 -712C>T polymorphism, it was observed that the frequencies of CT and TT genotypes were reduced in patients diagnosed in chronic phase as compared to controls, while the frequency of TT genotype was elevated in patients with accelerated phase. However, small sample size in these groups (accelerated and blast phase) does not permit to draw inference. The genotype distribution with respect to CASP9 -293 del and CASP9 Ex5 +32G>A polymorphisms did not reveal significant association (Table 7).

**Table 7:** Genotype distribution of CASP9 polymorphisms with respect to phase of CML.

CASP9 -1263A>G		AA	AG	OR (95% CI)	GG	OR (95% CI)	p-Value
	Controls	151 (29.61)	214 (41.96)	1.00	145 (28.43)	1.00	0.00008*
At the time of diagnosis	Chronic	71 (16.9)	200 (47.5)	**1.99 (1.41–2.80)**	150 (35.6)	**2.20 (1.53–3.16)**	
	Accelerated	4 (12.9)	16 (51.6)	2.82 (0.93–8.60)	11 (35.5)	2.86 (0.89–9.20)	
	Blast	1 (6.2)	9 (56.2)	6.35 (0.0–50.6)	6 (37.5)	6.25 (0.74–52.54)	
Progressed (chronic phase)	Without progression	46 (18.33)	114 (45.42)	**1.75 (1.17–2.61)**	91 (36.25)	**2.06 (1.35–3.14)**	
	With progression	25 (14.71)	86 (50.59)	**2.43 (1.4–3.97)**	59 (34.71)	**2.46 (1.46–4.14)**	

CASP9 -712C>T		CC	CT	OR (95% CI)	TT	OR (95% CI)	p-Value
	Controls	407 (80.12)	69 (13.58)	1.00	32 (6.30)	1.00	0.000006* 0.00007* (Yates)
At the time of diagnosis	Chronic	382 (90.7)	24 (5.7)	**0.37 (0.22–0.60)**	15 (3.6)	**0.50 (0.27–0.94)**	
	Accelerated	24 (80)	1 (3.3)	0.25 (0.03–1.85)	5 (16.7)	2.65 (0.95–7.41)	
	Blast	13 (81.2)	2 (12.5)	0.90 (0.20–4.10)	1 (6.2)	0.9 (0.12–7.71)	
Progressed (chronic phase)	Without progression	229 (91.24)	13 (5.18)	**0.33 (0.18–0.62)**	9 (3.59)	0.50 (0.23–1.07)	
	With progression	153 (90.00)	11 (6.47)	**0.42 (0.22–0.82)**	6 (3.53)	0.50 (0.20–1.21)	

CASP9 -293 del		+/+	+/−	OR (95% CI)	−/−	OR (95% CI)	p-Value
	Controls	121 (23.87)	231 (45.56)	1.00	155 (30.57)	1.00	0.40
At the time of diagnosis	Chronic	120 (28.7)	164 (39.2)	0.72 (0.51–0.9)	134 (32.1)	0.87 (0.62–1.23)	
	Accelerated	10 (34.5)	10 (34.5)	0.52 (0.21–1.29)	9 (31)	0.70(0.22–1.78)	
	Blast	5 (31.2)	4 (25)	0.42 (0.11–1.59)	7 (43.8)	1.09(0.33–3.52)	
Progressed (chronic phase)	Without progression	77 (30.92)	94 (37.75)	0.64 (0.44–0.92)	78 (31.33)	0.79(0.53–1.17)	
	With progression	43 (25.44)	70 (41.42)	0.5 (0.55–1.33)	56 (33.14)	1.01 (0.64–1.61)	

CASP9 Ex5 +32G>A		GG	GA	OR (95% CI)	AA	OR (95% CI)	p-Value
	Controls	83(16.40)	261(51.58)	1.00	162(32.02)	1.00	0.89
At the time of diagnosis	Chronic	67 (16.2)	218 (52.7)	1.03 (0.71–1.49)	129 (31.2)	0.98 (0.66–1.46)	
	Accelerated	6 (20)	15 (50)	0.80 (0.30–2.11)	9 (30)	0.77 (0.26–2.23)	
	Blast	0 (0)	9 (56.2)	6.07 (0.35–105.3)	7 (43.8)	7.71 (0.43–136.6)	
Progressed (chronic phase)	Without progression	44 (17.67)	127 (51.00)	0.92 (0.60–1.40)	78 (31.33)	0.91 (0.58–1.43)	
	With progression	23 (13.94)	91 (55.15)	1.26(0.75–2.11)	51 (30.91)	1.13 (0.65–1.99)	

OR- odds ratio adjusted by age and sex; p value- χ^2^ p value; *p < 0.05; ^#^p < 0.10.

### 3.4 Association of CASP9 polymorphisms with respect to IM resistance (primary and secondary)

The genotype data of chronic phase patients was further analyzed with respect to primary or secondary resistance. It was observed that the CASP9 -1263AG and CASP9 -1263GG genotypes had significantly increased the risk by several folds for both primary and secondary resistance, while the distribution of CASP9 -712C>T and CASP9 -293 del polymorphisms did not reveal any significant association. However, the AA genotype of CASP9 Ex5 +32G>A polymorphism was significantly associated with increased risk for secondary resistance (AA- OR-3.54, 95% CI- 1.02–12.22) (Table 8).

**Table 8:** Genotype distribution of CASP9 polymorphisms among primary and secondary resistant CML cases and controls.

CASP9 -1263A>G	AA	AG	OR (95% CI)	GG	OR (95% CI)	p-Value
Controls	151(29.61)	214(41.96)	1.00	145(28.43)	1.00	0.003*
Primary resistant cases	6 (11.76)	28 (54.90)	**3.29 (1.33–8.15)**	17 (33.33)	**3.01 (1.15–7.89)**	
Secondary resistant cases	4 (9.76)	18 (43.90)	**3.18 (1.05–9.58)**	19 (46.34)	**4.82 (1.59–14.60)**	

CASP9 -712C>T	CC	CT	OR (95 CI)	TT	OR (95 CI)	p-Value
Controls	407 (80.12)	69 (13.58)	1.00	32 (6.30)	1.00	0.36 0.58 (yates)
Primary resistant cases	44 (86.3)	4 (7.8)	0.54 (0.19–1.58)	3 (5.9)	0.87 (0.26–2.95)	
Secondary resistant cases	36 (85.71)	2 (4.76)	0.31 (0.07–1.31)	4 (9.52)	1.39 (0.46–4.15)	

CASP9 -293 del	+/+	+/−	OR (95 CI)	−/−	OR (95 CI)	p-Value
Controls	121 (23.87)	231 (45.56)	1.00	155 (30.57)	1.00	0.45
Primary resistant cases	10 (20.00)	23 (46.00)	1.20 (0.55–2.60)	17 (34.00)	1.33 (0.59–3.01)	
Secondary resistant cases	9 (21.95)	14 (34.15)	0.82 (0.34–1.95)	18 (43.90)	1.54 (0.67–3.56)	

CASP9 Ex5 +32G>A	GG	GA	OR (95 CI)	AA	OR (95 CI)	p-Value
Controls	83 (16.40)	261 (51.58)	1.00	162 (32.02)	1.00	0.13
Primary resistant cases	7 (14.58)	26 (54.17)	1.18 (0.49–2.81)	15 (31.25)	1.10 (0.43–2.81)	
Secondary resistant cases	3 (7.32)	17 (41.46)	1.81 (0.52–6.33)	21 (51.22)	**3.54 (1.02–12.22)**	

OR- odds ratio adjusted by age and sex; p value- χ^2^ p value; *p < 0.05; ^#^p < 0.10.

### 3.5 Association of CASP9 polymorphisms with the survival rate (event free and five overall survival rate)

**Fig. 2:**
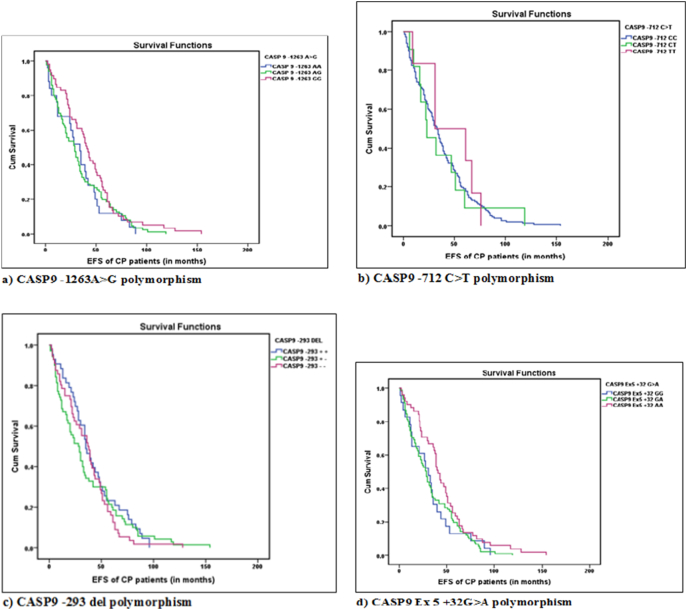
Kaplan Meier Survival curves for event free survival (EFS) rate with respect to Caspase 9 polymorphisms.

**Fig. 3:**
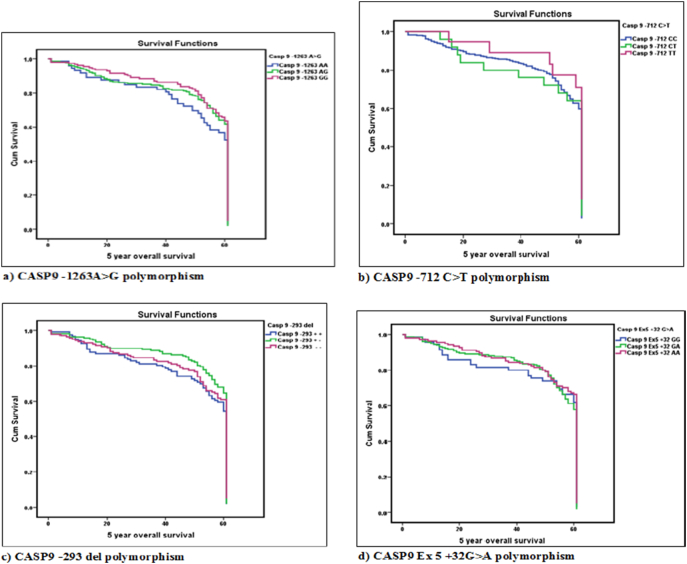
Kaplan Meier Survival Curves for five year overall survival (OS) rate with respect to Caspase 9 polymorphisms.

Kaplan Meier analysis of both event free and five year overall survival rates had failed to reveal significant association of CASP9 -1263G>A, CASP9 -712C>T, CASP9 -293 del and CASP9 Ex5 +32 G>A polymorphisms with CML (Table 9 and Fig. 2, Table 10 and Fig. 3).

**Table 9:** Kaplan Meier Survival analysis for event free survival (EFS) rate with respect to CASP9 polymorphisms.

Sl no	CASP9	Chronic phase (%)	(EFS in months) mean ± SEM	Median	p-Value
*CASP9 -1263 A>G*					
1	AA	25 (14.71)	33.20 ± 4.95	34.00	0.17^a^
2	AG	86 (50.59)	33.97 ± 2.96	29.00	
3	GG	59 (34.70)	43.83 ± 3.93	41.00	
Total		**170**	**37.28 ± 2.17**	**32.00**	

*CASP9 -712 C>T*					
1	CC	153 (90.00)	36.99 ± 2.28	33.00	0.86^a^
2	CT	11 (6.47)	36.64 ± 9.78	23.00	
3	TT	6 (3.53)	45.83 ± 10.62	31.00	
Total		**170**	**37.28 ± 2.17**	**32.00**	

*CASP9 -293 DEL*					
1	+/+	43 (25.44)	41.40 ± 4.02	35.00	0.67^a^
2	+/−	70 (41.42)	35.73 ± 3.87	28.00	
3	−/−	56 (33.14)	36.50 ± 3.26	37.00	
Total		**169**	**37.43 ± 2.18**	**33.00**	

*CASP9 Ex5 +32G>A*					
1	GG	23 (13.94)	32.74 ± 5.48	31.000	0.08^a^
2	GA	91 (55.15)	34.10 ± 2.80	29.000	
3	AA	51 (30.91)	46.06 ± 4.35	41.000	
Total		**165**	**37.61 ± 2.21**	**33.000**	

a) Log Rank (Mantle Cox) p value; p value- χ^2^ p value; *p < 0.05; ^#^p < 0.10.

**Table 10:** Kaplan Meier Survival analysis for five year overall survival (OS) rate with respect to CASP9 polymorphisms.

Sl no		N (%)	Chronic phase (%)	(OS in months) mean ± SEM	Median	p-Value
*CASP9 -1263A>G*						
1	AA	74 (16.41)	69 (93.24)	49.78 ± 2.07	61.000	0.19^a^
2	AG	221 (49.00)	197 (89.14)	51.88 ± 1.16	61.000	
3	GG	156 (34.59)	143 (91.67)	53.48 ± 1.20	61.000	
Total		**451**	**409 (90.69)**	**52.09** **±** **0.78**	**61.000**	

*CASP9 -712 C>T*						
1	CC	403 (89.56)	371 (92.06)	51.93 ± 0.83	61.000	0.37^a^
2	CT	27 (6.00)	24 (88.89)	50.56 ± 3.47	61.000	
3	TT	20 (4.44)	14 (70.00)	55.42 ± 2.96	61.000	
Total		**450**	**409 (90.89)**	**52.00** **±** **0.78**	**61.000**	

*CASP9 -293 del*						
1	+/+	128 (28.70)	115 (89.84)	50.00 ± 1.61	61.00	0.18^a^
2	+/−	172 (38.57)	159 (92.44)	53.84 ± 1.15	61.00	
3	−/−	146 (32.73)	132 (90.41)	51.37 ± 1.42	61.00	
**Total**		**446**	**406 (91.03)**	**51.95** **±** **0.79**	**61.00**	

*CASP9 Ex5 +32G>A*						
1	GG	71 (16.03)	65 (91.55)	50.57 ± 2.19	61.000	0.25^a^
2	GA	234 (52.82)	211 (90.17)	52.48 ± 1.05	61.000	
3	AA	138 (31.15)	126 (91.30)	52.60 ± 1.33	61.000	
Total		**443**	**402 (90.74)**	**52.33 ± 0.78**	**61.000**	

a) Log Rank (Mantle Cox) p value; p value- χ^2^ p value; *p < 0.05; ^#^p < 0.10.

### 3.6 Haplotype analysis of CASP9 polymorphisms

The haplotype analysis of all four CASP9 SNPs including 3 promoter SNPs and one exonic SNP (CASP9 -1263 A>G, CASP9 -712 C>T, CASP9 -293 del and CASP9 Ex5 +32G>A) was carried out to find out the risk haplotypes for CML development. Out of 10 haplotypes obtained, it was observed that two haplotypes G-C-del (+)-G (OR-1.61, 95% CI-0.97–2.65, p- 0.06^#^) and G-C-del(−)-G (OR-2.09, 95% CI-0.94–4.66, p- 0.07^#^) haplotypes were found to be associated with increased risk for the development of CML, while A-C-del (−)-A (OR-0.33, 95% CI- 0.20–0.55, p - <0.0001*) and A-T-del (+)-G (OR-0.32 (0.15–0.69), p- 0.004*) were significantly associated with decreased risk for the development of CML (Table 11).

**Table 11:** Haplotype analysis of promoter polymorphisms (CASP9 -1263 A>G, CASP9 -712 C>T, CASP9 -293 del) and exonic (CASP9 EX+5 32G>A) among cases and controls.

SL no.	CASP9 -1263 A>G	CASP9 -712 C>T	CASP9 -293 del	CASP9 1207 G>A	Frequency			OR (95% CI)	p-Value
					Total	Controls	Cases		
1	G	C	−	A	0.3629	0.3396	0.3876	1.00	−
2	A	C	+	G	0.298	0.3071	0.2901	0.87 (0.69–1.10)	0.25
3	G	C	+	A	0.0583	0.0456	0.0715	1.23 (0.86–1.76)	0.26
4	A	C	−	A	0.0562	0.0832	0.0279	**0.33 (0.20–0.55)**	<0.0001*
5	G	T	−	A	0.0484	0.0583	0.0399	0.72 (0.47–1.11)	0.14
6	G	C	+	G	0.0429	0.0309	0.0543	**1.61 (0.97–2.65)**	**0.06**^**#**^
7	A	C	+	A	0.0344	0.0296	0.039	1.08 (0.65–1.78)	0.78
8	A	T	+	G	0.027	0.0415	0.0117	**0.32 (0.15–0.69)**	0.004*
9	A	C	−	G	0.026	0.0232	0.029	1.01 (0.55–1.87)	0.97
10	G	C	−	G	0.0174	0.3396	0.0248	**2.09 (0.94–4.66)**	**0.07**^**#**^

Global haplotype association p-value: <0.0001; OR- odds ratio adjusted by age and sex; p value- χ^2^ p value; *p < 0.05; ^#^p < 0.10.

### 3.7 Linkage disequilibrium analysis of CASP9 polymorphisms

**Fig. 4:**
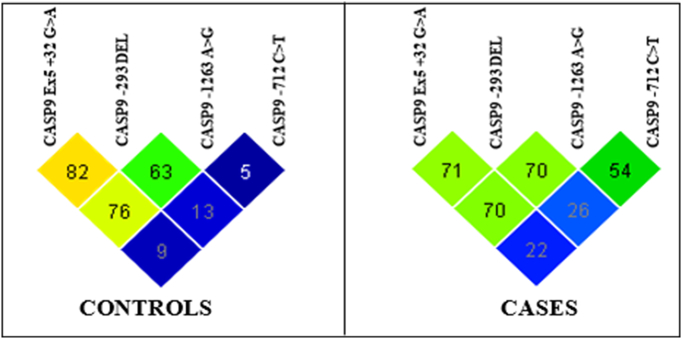
LD plot of Caspase 9 SNPs in controls and cases.

In the present study, pairwise LD analysis was performed for 4 SNPs independently in both cases and controls. It was observed that the all the four SNPs of CASP9 gene were found to be associated with either high or moderate LD in both cases and controls. CASP9 Ex5 +32 G>A and CASP9 -1263A>G exhibited high LD in both controls (D′-0.769) and cases (D′-0.702) while the SNP combinations of CASP9 Ex5 +32 G>A and CASP9 -293 del showed strong LD in controls (D′- 0.824) but moderate LD in cases (0.714). Further, comparison of LD values of CASP9 -293 del and CASP9 -1263A>G had shown moderate LD in controls (0.633) while high LD in cases (0.707). However, in case of CASP9 -1263A>G and CASP9 -712C>T SNPs, a moderate LD was presented in cases (0.547) while was absent in controls (0.051) (Table 12, Table 13 and Fig. 4).

**Table 12:**
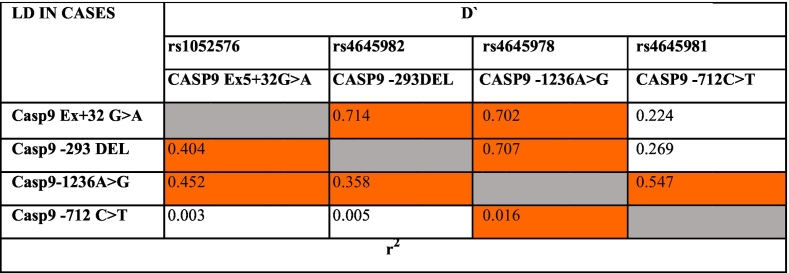
Pairwise LD for Caspase 9 polymorphisms studied in cases.

Strength of LD between any two markers is represented by the intensity of color box.

Red color: Higher probability(closer to 1); White: Probability Approaches random (0.5); Blue: Slightly higher than random; Light red: Between red and blue.

**Table 13:**
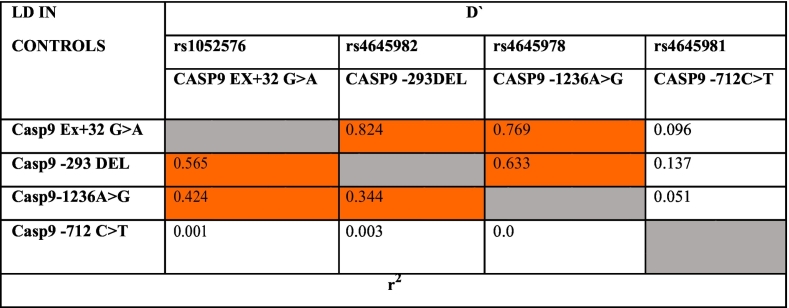
Pairwise LD for Caspase 9 polymorphisms studied in controls.

Strength of LD between any two markers is represented by the intensity of color box.

Red color: Higher probability(closer to 1); White: Probability Approaches random (0.5); Blue: Slightly higher than random; Light red: Between red and blue.

## 4 Discussion

Caspase 9 is the initiator caspase involved in mitochondrial-mediated/intrinsic apoptotic pathway. It is encoded by CASP9 gene which is located on chromosome 1 at p36.21 position (Hadano et al., 1999). Human CASP9 gene consists of 9 exons and 8 introns and spans over 35 kb region. Caspase 9 plays an important role in apoptosis and is thought to act as tumor suppressor gene. Initially, Caspase 9 exists as inactive proenzyme which undergoes proteolytic cleavage at conserved aspartic residues (Asp315 and Asp330) to produce large and small sub-units which further dimerize into active form. Although, several SNPs have been reported, four SNPs namely CASP9-1263G>A, CASP9 -712 C>T, CASP9 -293 del (−293 to -275 del CGTGAGGTCAGTGCGGGG A)) and CASP9 Ex5 +32G>A are of particular interest as the presence of these SNPs in the promoter and exonic region are known to influence the expression of the gene. Hence, in the present study, the association of these SNPs (promoter and exonic) with the development and progression of CML was analyzed.

The results of the present study with respect to CASP9 -1263G>A polymorphism had revealed that CASP9 -1263AG/GG genotypes and G allele might confer risk for the development of CML which was in accordance with case-control studies on breast cancer by Theodoropoulos et al. (2012) who reported individuals with CASP9 -1263AG or CASP9 -1263GG genotypes were at higher risk for breast cancer. Studies on other cancers like pancreatic cancer and papillary thyroid carcinoma also revealed the increased risk associated with the G allele (Theodoropoulos et al., 2010; Wang et al., 2012). However, contrary results were obtained from the studies on other cancers like lung (Park et al., 2006), bladder (Gangwar et al., 2009), prostate (Kesarwani et al., 2011) and gastric cancer (Liamarkopoulos et al., 2011) as well as meta-analysis of 9 different studies (prostate) which revealed reduced risk associated with G allele to develop cancer in Asian population (Xu et al., 2012a). The increased frequency of AG and GG genotypes irrespective of phase of CML and elevated frequency of AG genotype in chronic CML patients with subsequent progression indicated possible role of SNP in CML development and progression. Significant association of GG genotype with both primary and secondary resistance further confirmed the importance of this SNP in determining Imatinib response.

The results with respect to CASP9 -712C>T polymorphism suggested that the presence of CASP9 -712T allele was associated with the reduced risk for the development of CML. Previous studies on transcription factor binding sites using Alibaba2 programme had revealed the loss of binding sites for Krox-20, NF-1, and ETF transcription factors in the presence of -712 T allele resulting in reduced promoter activity and decreased apoptosis (Park et al., 2006). The results of the present study were in disagreement with the earlier reports on lung cancer (Park et al., 2006; Lee et al., 2010), breast cancer (Theodoropoulos et al., 2012), Non-Small Cell Lung Cancer (Javid et al., 2013) and AML (Cingeetham et al., 2015) where the T allele was found to be significantly associated with increased risk which was also supported by meta-analysis studies, where the population based stratification of the data revealed significant risk associated with T allele to develop lung cancer (Xu et al., 2012a). It may be possible that T allele as such may not offer protection against CML in view of the reduced apoptosis associated with the allele, but may appear to do so by being in equilibrium with other promoter SNPs favoring apoptosis.

Presence of heterozygote CASP9 -293 del (+/−) genotype may confer protection against CML as confirmed by the present case-control study. The results obtained in the present study were in agreement with the study by Gangwar et al. (2009), who reported protective effect exerted by heterozygous (−/+) genotype in high-risk non-muscle-invasive bladder cancer (NMIBC). However, studies on lung (Park et al., 2006), prostate cancer (Kesarwani et al., 2011) and AML (Cingeetham et al., 2015) failed to reveal association of CASP9 -293 del polymorphism with cancer risk. CASP9 -293 del (+/−) genotype conferred protection against CML risk as evidenced from observation made with respect to phase of the disease and IM response, while the homozygous CASP9 -293 del (−/−) genotype conferred increased risk for the progression as observed by the elevated frequency of CASP9 -293 del (−/−) genotype in patients diagnosed in blast crisis and in secondary resistance cases. These results therefore indicated that the presence of a single dose of a normal CASP9 -293 del (+) allele in heterozygotes might be able to compensate the risk conferred by deletion allele, whereas deletion of 19 nucleotides in the promoter region, on both chromosomes (−/−) might affect the activity of CASP9, there by apoptosis.

In CASP9 Ex5 +32G>A polymorphism, the transition of G allele with A leads to a conformational change in the structure of Caspase 9 which in turn could modify the affinity of Caspase 9 protein to Apaf-1, thereby affecting the apoptotic machinery. Although several studies have reported the association of CASP9 Ex5 +32G>A polymorphism with cancer susceptibility, the results of present study however, failed to reveal significant association of this polymorphism with the development and progression CML. The results of the present study were in agreement with earlier studies on lung cancer (Xu et al., 2012b) and AML (Cingeetham et al., 2015). However, contradictory results were reported in the studies carried out on women with non-Hodgkin lymphoma (Lan et al., 2007) and multiple myeloma (Hosgood et al., 2008) where the variant ‘A’ allele was found to be associated with decreased risk. The decreased risk associated with A allele was further supported by two independent meta-analysis studies carried out on several cancers (Xu et al., 2012a; Yan et al., 2013). Although significant association of CASP9 Ex5 +32G>A polymorphism was not observed with the development of CML in the present study, it was observed that the CASP9 Ex5 +32AA genotype had increased the risk to develop secondary resistance which indicated the possibility of interaction between variant genotype and other Bcr/Abl dependent or independent mechanisms.

There was no significant association of CASP9 polymorphisms with mean EFS and relative five year overall survival rates. Haplotype analysis performed to find out the association of CASP9 -1263 A>G, CASP9 -712 C>T, CASP9 -293 del and CASP9 Ex5 +32G>A with the development of CML had confirmed that the presence of variant G allele of CASP9 -1263 A>G might be independently conferring risk for the development of CML. The presence of strong LD observed between SNPs of CASP9 genes indicated that these promoter SNPs tend to move together because of their close physical vicinity. However, the presence of LD in cases and absence in controls as observed in the case of SNP combination of CASP9 -1263A>G and CASP9 -712C>T might be because of the hitchhiking effect, a phenomenon where an allele changes its frequency not because of its natural selection, but, because of its presence near another SNP, which is undergoing a selective sweep.

## 5 Conclusion

The results of the present study concludes that the G allele of CASP9 -1263A>G polymorphism might confer risk for the development and progression of CML while CASP9 -712C>T and CASP9 -293 del SNPs might confer protection against development of CML. The presence of CASP9 -293 del (−/−) and CASP9 Ex5 +32 AA genotypes might confer risk for the progression.

## Author contributions

We would like to thank all the CML patients and volunteers who gave consent to participate in the study. PME contributed in sample collection, acquisition of data, genotyping of the samples, data analysis, interpretation of data and writing manuscript, MG and SK contributed in sample and data collection from the patients, AC collected control samples; VS designed and conceived the study, supervised experiments, data analyses and critical revision of the manuscript, AS contributed in proof reading the manuscript; RD contributed in providing clinical data.

## Funding

PME is grateful to the Council of Scientific and Industrial Research - Extra Mural Research (CSIR-EMR-II) project (vide no-027/(0258)/12/EMR-II) and Osmania University-Department of Science and Technology (OU-DST)-PURSE program, 10.13039/501100004207Osmania University for the financial assistance to carry out the research work and 10.13039/501100001411Indian Council of Medical Research (ICMR), New Delhi, for funding in the form of Senior Research Fellowship.

## Declaration of interest

None.
